# The Science and Art of Surgery

**Published:** 1873-04

**Authors:** 


*The Science and Art of Surgery. Being a Treatise on Surgical Injuries, Diseases, and Operations.* By JOHN ERIC ERICHSEN, Senior Surgeon to University College Hospital, and Holme Professor of Clinical Surgery in University College, London. A New Edition, illustrated by upwards of seven hundred Engravings. In two volumes. Philadelphia: Henry C. Lea, 1873. Buffalo: T. Butler & Son.

Erichsen's Surgery has long been considered as one of the best Surgical text books published, and the fact that six editions have been issued shows it to be duly appreciated by the profession. The present edition comes to us bound in two separate volumes, and is considerably increased in size over the last American edition.

It has been too long known by the medical profession to need any very extended notice or any critical review of its contents.

The first chapter is devoted to anæsthetics, and to a general consideration of and rules to be observed in preparing for operations.

The author seems to give his preference to chloroform, evidently not being much accustomed to the use of ether. Chapters two and three are taken up by a consideration of amputations, the rules which are given by the author are concise and do not confuse the reader by a confusion of methods; the best procedures and those which have stood the test of trial only being given. Inflammation, Suppuration, Ulceration and the Processs of Repair complete the first Division. The second Division treats of Surgical Injuries, the third of Surgical Diseases. The directions for diagnosis and treatment which are given by the author are such as may with safety be followed. Many of the more recent propositions in operative Surgery have been omitted, but they are as a general rule either too recent to have come to the author's knowledge, or of such doubtful value as to forbid their introduction into a work which aims to give the best known rules in as brief a space as is consistent with clearness. We are sorry however to see no mention of Prof. Moore's method of dressing Colles fracture and fracture of the Clavicle. The illustrations are good, although most of them are familiar. The work as presented is a valuable addition to the surgeons library, and is to be recommended to all who wish a work on surgery as one of the finest treatises published.

